# Complex Biological Reconstruction after Wide Excision of Osteogenic Sarcoma in Lower Extremities

**DOI:** 10.1155/2013/538364

**Published:** 2013-01-17

**Authors:** Kashif Abbas, Masood Umer, Haroon ur Rashid

**Affiliations:** ^1^Islam Medical College, Sialkot, Pakistan; ^2^H No. 88 K-1, Wapda Town, Lahore, Pakistan; ^3^Aga Khan University Hospital, Karachi, Pakistan

## Abstract

Wide margin resection of extremity tumor sometimes leaves a huge soft tissue and bony defects in limb salvage surgery. Adequate management of these defects is an absolute requirement when aiming for functional limb. Multidisciplinary management in such cases is an answer when complex biologic reconstruction is desired. We aim to present cases of osteogenic sarcoma of lower extremity requiring combined surgical approach to achieve effective musculoskeletal reconstruction. *Patients and Methods*. From 2006 to 2010 ten patients were operated on for osteogenic sarcoma of lower extremity requiring complex musculoskeletal reconstruction. *Results*. Six patients had pathology around knee joint, whereas one each with mid tibia, mid femur, proximal femur, and heel bone. Locking compression plate was used in 7 patients including six with periarticular disease. Eight out of ten patients underwent biologic reconstruction using autograft; endoprosthetic reconstruction and hindquarter amputation were done in the remaining two patients. Vascularized fibula was done in five patients, sural artery flap which was primarily done in three patients, spare part fillet flap, free iliac crest flap, and Gastrocnemius flap was done in one patient each. Secondary hemorrhage, infection, nonunion, wound dehiscence, and flap failure were notable complications in four patients. The Average Musculoskeletal Tumor Society score was 89%. *Conclusion*. Combined surgical approach results in cosmetically acceptable and functional limb.

## 1. Introduction

Osteogenic sarcoma is the most common primary malignant bone tumor in children and adolescents. Historically, more radical treatment options were employed in the management of these tumors in the form of amputation or disarticulations. With advances in multidisciplinary approach toward management, over 90% are limb preserving surgeries. Options for limb salvage reconstruction after wide resection include osteoarticular allograft, allograft prosthetic composite, recycled autograft, and modular or custom made endoprosthesis [1–3].

Due to financial constraints, biologic reconstruction with recycled autograft is common in our part of the world. The long-term result in terms of joint range of motion (ROM) is as good as endoprosthetic reconstruction. However, it requires immobilization during immediate postoperatively till the time of union.

Conventionally, wide margin resection is an absolute requirement to ensure adequate resection in order to decrease the risk of disease recurrence. However with recent advances in surgical oncology, more conservative resections have been proposed near vital regions [4, 5]. 

The most heroic and beautifully performed vascular and bony reconstructions are wasted without concomitant soft tissue coverage of these repairs. Suboptimal coverage can lead to prosthesis infection, subsequent hardware exposure, or loss with eventual amputation. The strategy of meticulous resection as well as reconstruction of bone and soft tissue works together for optimum results. This strategy may require additional microvascular skills on part of a single surgeon or two surgeons can work together as a team. 

The aim of the current study is to present our cases of osteogenic sarcoma of lower limb requiring combined surgical approach to achieve effective musculoskeletal reconstruction. 

## 2. Materials and Methods

This is a retrospective review of ten patients who underwent reconstruction of oncologic defects at our institution from 2006 to 2010. All patients with osteogenic sarcoma of lower extremities requiring combined musculoskeletal and soft tissue reconstruction for wound closure in index surgery were included. We excluded cases that required split or full thickness skin grafting as a sole means of wound coverage. Any patients who underwent flap surgery as a result of initial wound related complications were also excluded. Medical record number is retrieved through surgical team database and demographics and further details were reviewed through confidential files and hospital based software called Patient Care Inquiry (PCI), containing patient records of hospital visits. The tumor length, width, and depth were measured based on sagittal, coronal, and axial magnetic resonance imaging (MRI). Primary outcomes, that is, uptakes of the flap, were evaluated. Perioperative complications were also noted including donor as well as recipient sites. Functional outcome was assessed using Musculoskeletal Society Tumor Score. 

## 3. Surgery 

Our surgery team comprises two surgeons; each specialized in tumor surgery and soft tissue reconstruction. All surgical, metastatic workup and baseline investigations were done in outpatient setting. In nine, tissue diagnosis is confirmed by biopsies done in outpatient setting under local anesthesia. In one of our patients with recurrent disease biopsy was done in operating room from proximal femur in general anesthesia; rest of the work up was on the same lines. Multidisciplinary care structure was followed in all patients. All patients received neoadjuvant chemotherapy. For surgery, patients got admitted a day prior to surgery for final preoperative assessment by anesthetist as well as surgical team.

All surgeries were done under general anesthesia. Preoperative dose of Tranexamic acid 1 g and cefazolin 1 gm is a routine at the time of induction. Most resections were done under tourniquet. We routinely send frozen section of tissue or bone marrow from all margins after wide margin resection before embarking on final reconstructive procedure, bony as well as soft tissue. Reconstruction following tumor resection is done with new sets of instrument. Microsurgical aids were used where required. In few cases of vascularized fibula, the procedure (tumor resection and reconstruction) was started simultaneously with two different teams and different set of instruments to minimize surgical timing.

As a part of our protocol, the patient stays in the recovery room until becoming hemodynamically stable for two consecutive hours. Flap monitoring protocol includes assessment for capillary refill and color and warmth which is done on hourly basis for the initial 12 hours followed by 4 hourly monitoring. First dressing is usually done after 3 days. Followup is weekly for the first 3 weeks followed by monthly visit for the next 3 months. Patients are then followed up according to need. Patients living in remote cities are followed on phone and mail. 

## 4. Results 

During this period 10 patients underwent wide margin resection for osteogenic sarcoma followed by reconstruction. Seven patients were female. Mean age of patient was 18 yrs (12–40 yrs). Mean followup of 18 months is available. Six out of ten had pathology around knee joint, one with mid tibia, mid femur, proximal femur and calcaneum each. Skeletal reconstruction in periarticular tumor is relatively more challenging than diaphyseal tumor. We used locking compression plate in skeletal reconstruction of 7 patients including six with periarticular disease. Eight out of ten patients underwent biologic reconstruction using autograft; in addition three had autoclaved bone whereas the rest had fibula from the other leg mixed with corticocancellous graft. Synthetic bone granules of beta tricalcium phosphate were used in three patients. Endoprosthetic reconstruction and hindquarter amputation were done in the remaining two patients. Vascularized fibula was done in five patients to augment biologic reconstruction; out of this, one had this in addition to sural artery flap which was primarily done in three patients. Spare part fillet flap was done in a patient with proximal femur osteosarcoma after hind quarter amputation. Free iliac crest flap was done to reconstruct heel after tumor resection. gastrocnemius muscle flap was done in patients with extra-articular resection of knee joint to provide soft tissue cushion and vascularity (Table 1).

Complications included flap failure, reactive hemorrhage, and infection and wound dehiscence in each patient.

Bony union was noticed in all except one, who had undergone vascularized fibula and rigid fixation with longer plate.

All patients with tumor around knee joint showed no instability of the knee in the followup. All patients had no evidence of disease until the last followup. The MSTS functional outcome score was 89%.

## 5. Discussion 

Advances in the management of bone sarcoma have resulted in significant improvements in survival and quality of life [6, 7]. Several factors have likely contributed to these advances, including improved surgical technique and the development of referral centers for sarcoma treatment that have embraced a multidisciplinary approach [6]. The goal is to optimize oncologic outcome and maximize functional restoration. Reconstructive surgery after musculoskeletal sarcoma resection provides adequate coverage of wound, preserves function, and optimizes the cosmetic outcome. 

There are many methods that can be used to close excision defect. Primary closure is best for smaller defect. For slightly larger defects that are not amenable to primary closure, split thickness skin graft can be done if fascia or muscle is preserved [8]. In the case of long bone sarcoma resection, the resulting defect is usually large and complex and the traditional reconstruction is based on avascular allografts and local tissue flaps. However, allografts are associated with high rates of infection, nonunion, and fracture, leading to failure in about 50% of cases. Microvascular free flaps that contain bone such as free fibula flaps have been used instead of allografts with good success rates.

Defects of proximal third of the leg can usually be covered with medial or lateral gastrocnemius muscle or myocutaneous flap or a combination of the two. In our series we have done this flap to cover implants used for knee reconstruction. Wound dehiscence was noted within two weeks of surgery but fortunately implant was not directly exposed to the external environment. To overcome the feared complication of infection, free latissimus dorsi flap was done to cover the defects produced after wound dehiscence. Subsequent recovery of the patient was uneventful. 

**Figure 1:**
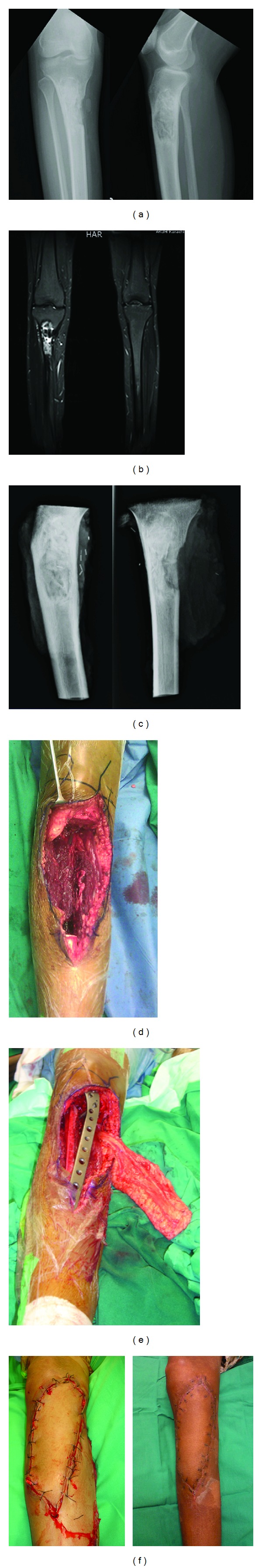
(a) Preoperative radiograph showing lesion in proximal tibia, (b) MRI showing exact dimension of signal changes in proximal tibia, (c) specimen radiograph taken intraoperatively, (d) Intraoperative picture showing defect after tumor excision, (e) reconstruction of defect with vascularized fibula and Locking compression plate and sural artery based myocutaneous flap, (f) immediate postoperative picture comparison with 2-week postoperative picture, (g) postoperative X-rays, (h) picture at 2 yrs of followup showing flap and donor site.

Sural flap coverage is classically done for defects around mid or distal tibia but in our series it was done for three cases of proximal tibia osteosarcoma (Figure 1). With slight modification in the technique and selection of donor site more proximally in the calf it is possible to extend the pedicle for proximal tibia defect coverage without additional flap related morbidities [9]. Bony reconstruction was done with the help of autograft nonvascularized fibula with corticocancellous graft. In one case vascularized fibula was done in addition to sural artery flap. 

Salvage of a nonfunctional limb is of little value for the patient. Likewise, patients with severe medical problems may not be good candidates for limb salvage procedures. In those situations, amputation of the lower extremity is indicated. Coverage should be enough to provide a good stump to fit an external prosthesis. Fillet flaps are harvested immediately and converted to flaps transferred to defect site. Studies show that they are oncologically safe and reliable [10]. In our series one patient had undergone hindquarter amputation for recurrent tumor. Fillet flap was done successfully without additional donor site morbidity. On the 4th postoperative day excessive drop in hemoglobin and expanding hematoma was noticed underneath the flap; thus he was rushed to the operating room for exploration where only generalized ooze was found without any gross evidence of infection; thus the wound closed again over drain. Subsequent recovery was uneventful without disease recurrence in 30 months of followup. 

The use of free fibular flap has been widespread since it was first described by Taylor in 1975 [11]. The presence of a free fibular flap to augment the construct does not provide strength to the overall construct. However it does appear to hasten the time to full weight bearing. Presence of free fibular flap appears to reduce the number of secondary procedures required.

In series using free fibular flaps as the sole modality, fibular union occurs in 74%–100% of cases reported. The incidence of delayed union is 16.7%–45%. The infection rates are 10%–15.4%. The stress fracture rates with free fibular flaps alone are 7.7%–22.2%. The overall complication rate with free fibular flaps alone is of the order 50%–54% [12].

In our series four patients had undergone vascularized fibula as an adjunct in the primary procedure to expedite biological reconstruction. One patient had delayed union in which 24 cm of ipsilateral vascularized fibula was used along with nonvascularized contralateral fibula, to fill the diaphyseal defect of tibia. Initial fixation device was removed and limb was put in cast for 2 months following healing. Full weight bearing is not yet allowed as the tibialization of fibulas is not complete in this case. In the rest of the cases vascularized fibula was used as onlay graft; thus good healing was evident with return to previous weight bearing status in an average of 11 months.

In one of the patients, heel defect was reconstructed with vascularized iliac crest flap which failed gradually. Her wound was managed with secondary intention with local control of infection with antibiotics and resection of sequestrum. She is now ambulating with a heel minus foot and has a well-adapted gait. 

## 6. Conclusion 

Combined surgical approach is an essential need especially when aiming for limb salvage. It gives an opportunity to ensure adequate surgical margins without fear of resultant wound defects and their coverage. Close liaison with histopathologist is also required to have margins status intraoperatively so that one stage reconstruction is carried out.

## Figures and Tables

**Table 1:** 

	Gender	Age (yrs)	Site	Biopsy	Surgery	Flap	Followup (months)	Status at last followup	Complication	MSTS functional score (%)
1	Male	40	Right neck of femur	Osteosarcoma	Hindquarter amputation	Fillet flap	30 months	NED	Secondary hemorrhage	70
2	Female	13	Left proximal tibia	Osteosarcoma	Wide margin excision	Sural artery flap	24 months	NED	Nil	95
3	Female	19	Right proximal tibia	Osteosarcoma	Wide margin excision	Tibialization + sural artery flap	24 months	NED	Nil	95
4	Female	20	Calcaneum mass	Osteosarcoma	WME	Free iliac crest flap	32	NED	Flap failure	88
5	Male	17	Right proximal tibia	Osteosarcoma	Wide margin excision	Sural artery flap	14	NED	Infection/nonunion	95
6	Female	20	Left distal femur mass	Osteosarcoma	Extra-articular resection of knee mass	Gastrocnemius flap/free latissimus dorsi flap	20	NED	Initial wound dehiscence	92
7	Female	14	Distal femur mass	Osteosarcoma	Wide margin excision	Vascularized fibula	14	NED	Nil	90
8	Female	15	Right mid tibia	Osteosarcoma	Wide margin resection	Vascularized fibula	12	NED	Nil	85
9	Female	13	Right mid femur mass	Osteosarcoma	Wide margin resection	Vascularized fibula	10	NED	Nil	92
10	Male	12	Distal femur lesion	Osteosarcoma	Wide margin resection	Vascularized fibula	10	NED	Nil	90
